# Formation and In Situ Treatment of High Fluoride Concentrations in Shallow Groundwater of a Semi-Arid Region: Jiaolai Basin, China

**DOI:** 10.3390/ijerph17218075

**Published:** 2020-11-02

**Authors:** Zongjun Gao, Mengjie Shi, Hongying Zhang, Jianguo Feng, Shaoyan Fang, Yechen Cui

**Affiliations:** College of Earth Science and Engineering, Shandong University of Science and Technology, Qingdao 266590, China; zongjungao1964@163.com (Z.G.); smj_sdust@126.com (M.S.); zhy10sdust@126.com (H.Z.); fsysdust@126.com (S.F.); cycsdust@126.com (Y.C.)

**Keywords:** fluoride formation, groundwater, in situ treatment, Jiaolai basin

## Abstract

Fluorine is an essential nutrient, and excessive or deficient fluoride contents in water can be harmful to human health. The shallow groundwater of the Jiaolai Basin, China has a high fluoride content. This study aimed to (1) investigate the processes responsible for the formation of shallow high-fluoride groundwater (SHFGW); (2) identify appropriate methods for in situ treatment of SHFGW. A field investigation into the formation of SHFGW was conducted, and the results of experiments using soils from high-fluoride areas were examined to investigate the leaching and migration of fluoride. The results showed that the formation of SHFGW in the Jiaolai Basin is due to long-term geological and evaporation processes in the region. Stratums around and inside the basin act as the source of fluoride whereas the terrain promotes groundwater convergence. The hydrodynamic and hydrochemical conditions resulting from slow groundwater flow along with high evaporation and low rainfall all contribute to the enrichment of fluoride in groundwater. In situ treatment of SHFGW may be an effective approach to manage high SHFGW in the Jiaolai Basin. Since soil fluoride in high-fluoride areas can leach into groundwater and migrate with runoff, the construction of ditches can shorten the runoff of shallow groundwater and accelerate groundwater loss, resulting in the loss of SHFGW from high-fluoride areas through river outflow. The groundwater level will be reduced, thereby lowering the influence of evaporation on fluoride enrichment in shallow groundwater. The results of this study can act a reference for further research on in situ treatment for high-fluoride groundwater.

## 1. Introduction

Humans depend on the natural environment for survival, and the quality of the natural environment directly affect the health of people [1]. Fluoride is an essential nutrient, and due to its active chemical properties, adopts various forms in the environment. Water-soluble fluoride has a considerable impact on human health, with excessive groundwater fluoride content often resulting in regions of endemic fluorosis [2]. Endemic fluorosis is a geochemical disease occurring in a specific geographical environment [3,4], and since its spatiotemporal distribution is positively correlated with the distribution of groundwater fluoride content, excessive fluoride content in groundwater is considered to be the main cause of regional fluorosis [5]. Groundwater is an important source of drinking water for human beings and is also the main source of irrigation water for agriculture in many areas. High groundwater fluoride contents can result in excessive uptake of fluoride into the human body through the direct consumption of drinking water, thereby posing a risk to human health [2,6,7,8,9,10,11]. In addition, the use of high-fluoride groundwater as irrigation water can lead to the build-up of excessive fluoride in crops, thereby posing a risk to the health of the society [12,13,14,15].

There have been many in-depth studies in recent decades on the problem of high fluoride in groundwater. Studies on the distribution of fluoride in groundwater have been conducted globally, with further investigation on the source and formation of fluoride in groundwater (Table 1) [7,16]. While fluorine is used in industrial processes for the manufacture of steel, aluminum and refrigerants, the majority of fluoride present in groundwater and surface water is of natural origin [17]. The different processes contributing to the formation of high-fluoride groundwater (HFGW) can be summarized as follows: (1) evaporative concentration, (2) leaching enrichment, (3) transgression enrichment, (4) hot water enrichment and (5) anthropogenic pollution [18,19,20,21,22]. HFGW areas formed though evaporative concentration are generally characterized by a flat or low-lying terrain, slow groundwater flow, less well-developed hydrodynamic conditions, shallow groundwater depth and high evaporation. Shallow groundwater under this specific supergene geochemical environment has high concentrations of fluoride and other chemical elements [23].

Various physical and chemical drinking water defluoridation methods have been employed to alleviate the problem of regional fluorosis resulting from high fluoride soil and groundwater [16,32,33]. Various adsorbates including alumina, bone char, brick piece columns and mud pots have been reported for removal of fluoride in water by adsorption [34,35,36]. Zhang and Huang [37] proposed the use of grape pomace as a biosorbent, which showed good adsorption capacity in experiments. The use of ion exchange for the removal of fluoride ions in water employs anionic and cationic exchange resins composed of synthetic chemicals [38]. Fluoride can also be removed from water by being precipitated in an insoluble form [39,40]. Membranes with specific pore size and compound-specific permeability are used for defluoridation, and this process includes reverse osmosis, nanofiltration through a membrane and electrodialysis [41]. Phytoremediation is being considered as an effective and low-cost remediation technique for the decontamination of soils [42,43,44]. Apart from these physical and chemical methods, biological processes for phytoremediation and defluoridation of soil, water or air using a bio-sorbent prepared from plant material and bioremediation through microbes have also been reported [41]. Fluoride-tolerant bacteria play an important role in the bioremediation and biotransformation of fluoride into a form that is less bioavailable and effectively reduce fluoride by facilitating the binding of fluoride to ions [45]. At present, effective measures adopted for managing areas of endemic fluorosis include the replacement [46] or treatment of the water source by adsorption, ion exchange, electrochemistry and other means [47]. These measures have reduced the fluoride content of drinking water, thereby effectively controlling the phenomenon of fluorosis [48]. However, the fluoride content in soil and groundwater of high-fluoride areas remains high, and the irrigation of crops using local high-fluoride waters continues [49].

The Jiaolai Basin is a typical shallow high-fluoride groundwater (SHFGW) distribution area situated in Northern China. The local shallow groundwater fluoride concentration in this region ranges from 0.02 mg L^−1^ to 25 mg L^−1^, which poses a serious health risk to residents. In an effort to control this risk, the local government has conducted extensive research to identify various water treatment methods for effectively reducing the fluoride content in drinking water and regulating the low-fluoride water sources in neighboring areas. Although these efforts have led to an improvement of the health status of residents [50], there has been no significant change in the fluoride content of shallow soil and groundwater, and some impacts on human health remain. Therefore, a reduction in the fluoride content of soil and groundwater is required to fundamentally remove the risk of high-fluoride soil and groundwater to human health.

The current study aimed to: (1) investigate the processes responsible for the formation of shallow high-fluoride groundwater (SHFGW) and (2) identify appropriate methods for in situ treatment of SHFGW. To achieve these aims, the current study conducted a hydrogeological investigation on the distribution of SHFGW in the Jiaolai Basin. In addition, the current study conducted soil leaching experiments in a laboratory using a soil column and soil tank. The results of the present study can provide a theoretical basis for subsequent research on in situ treatment methods for reducing fluoride content in soil and groundwater.

## 2. Materials and Methods

### 2.1. Study Area

#### 2.1.1. Geological Structure

**Figure 1:**
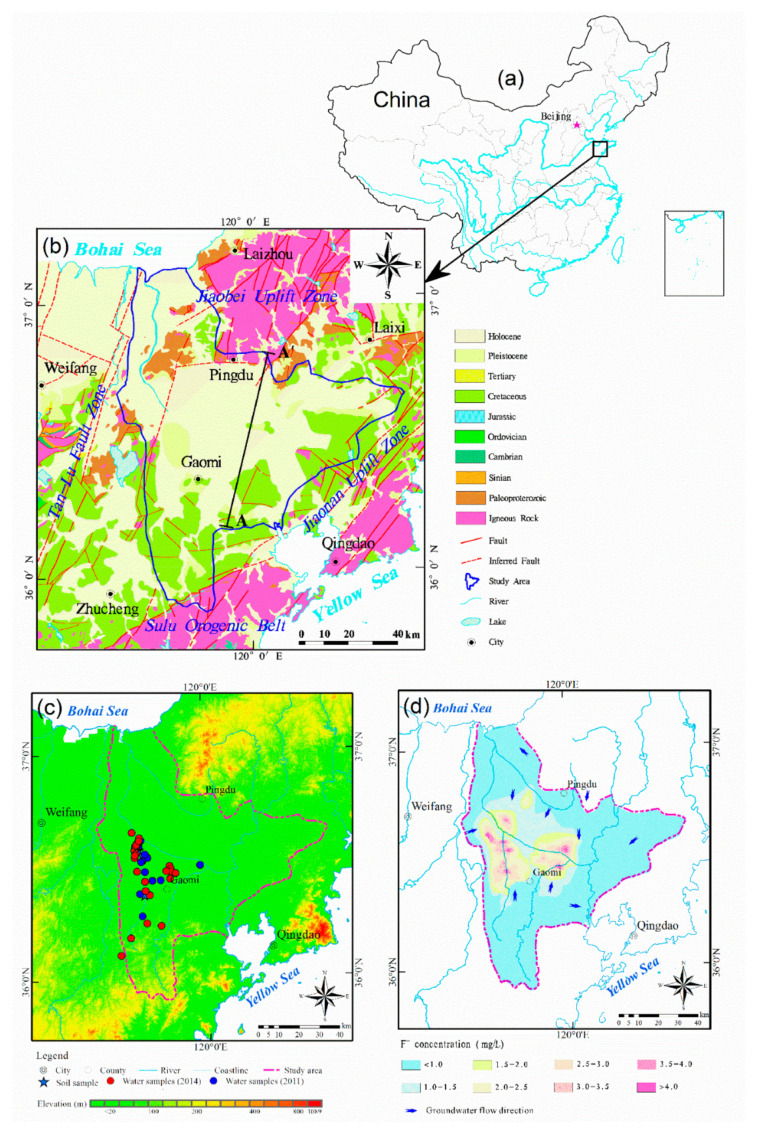
Survey of the Jiaolai Basin, Jiaodong Peninsula, eastern China. (**a**) location of study area, (**b**) stratigraphy and geological structures [51,52,53], (**c**) geomorphology and locations of water and soil samples, (**d**) fluoride distribution in groundwater.

The Jiaolai Basin is located in the Jiaodong Peninsula, eastern China (Figure 1). The region contains Cretaceous faults and experiences multi-stage tectonic evolution and transformation and is superimposed by different basin prototypes [51]. The western boundary of the Jiaolai Basin is the Yishu Fault Valley System located in the central Tanlu Fault Zone; the southern boundary is the Sulu Orogenic Belt, and the northern and eastern boundaries are the Jiaobei Uplift Belt and the Jiaonan Uplift Belt, respectively (Figure 1b). The unique geotectonic characteristics of Jiaolai Basin have for a long time attracted the attention of geologists [52].

#### 2.1.2. Geomorphology and Stratigraphy

The geomorphological types present in the Jiaolai Basin include low and sloping hills, ramp denudation plain and low flat alluvial plain. Low hills are distributed around the basin, with the Jiaolai alluvial plain located in the center and topographic inclines moving from the surrounding to the center [53]. Gneiss and marble strata of the Jiaodong and Fenzishan groups of the Archean and Proterozoic are exposed in the northeast, whereas sandstone and conglomerate of the Qingshan and Wang’s groups of the Cretaceous are exposed in the west and south. The interior of the basin is covered with Quaternary alluvial strata, characterized by a mainly clay and sub-clay lithology with slow groundwater flow (Figure 1b). Table 2 shows the main stratum and lithology features of the Jiaolai Basin.

#### 2.1.3. Meteorology and Hydrology

The Jiaolai Basin is located within the middle latitudes and falls within a semi-humid monsoon climate zone characterized by four distinct seasons with lower rainfall. Annual average precipitation and evaporation ranges from 652 mm to 821 mm and 1721 mm to 1984 mm, respectively [53]. The majority of rivers in the basin originate from the surrounding mountainous areas, with three seasonal river systems being the Dagu, North Jiaolai and South Jiaolai rivers [23]. Groundwater in the Jiaolai Basin can be divided into four categories: (1) pore water, (2) pore fissure water, (3) karst water and (4) bedrock fissure water. The loose rock pore water (1) constitutes the dominant type [56]. Groundwater is mainly recharged by precipitation, and runoff and discharge vary with topography, landform and artificial exploitation. Groundwater tends to be shallow, with precipitation and exploitation being the main factors directly controlling changes in groundwater level [49].

#### 2.1.4. Distribution of Fluoride in Groundwater

The groundwater fluoride concentration in the Jiaolai Basin ranges from 0.2 mg·L^−1^ to 16 mg·L^−1^. Approximately 75.27% of the area has a groundwater fluoride concentration <1.0 mg·L^−1^, with this zone widely distributed along the margin of the study area. Approximately 24.14% of the study area has a groundwater fluoride concentration ranging from 1.0 mg·L^−1^ to 3.0 mg·L^−1^, mainly distributed in the center and western parts. Only 0.59% of the study area has a groundwater F^−^ concentration exceeding 3.0 mg·L^−1^, mainly distributed in the low-lying plains along both sides of the Jiaolai River in the central part (Figure 1d) [23,53,56,57].

### 2.2. Sampling Materials and Methods

The present study collected water and soil samples mainly from the high-fluoride areas south of the Jiaolai River. A total of 44 groundwater samples were collected during the rainy season from July to September in 2011 and 2014 (Figure 1c). Groundwater samples were collected using sterilized polythene plastic bottles with a volume of 2 L. Soil samples were collected at 20 cm intervals and at 80 cm depth. Two groups of ten soil samples were collected in the villages of Nanxiegou and Zhoujiazhuang. In addition, soil samples were collected from the village of Damoujia for the laboratory-based leaching experiments. Those soil samples were divided into five categories according to layer depth in the range of 0 to 80 cm, numbered as T_1_, T_2_, T_3_, T_4_ and T_5_, with the thickness of each layer from top to bottom being 10, 22, 23, 15 and 10 cm, respectively.

### 2.3. Experimental Design and Procedure

Experiments conducted in the present study included two components: the soil column leaching experiment and soil tank leaching experiment. Both experiments involved leaching tests on in situ high-fluorine soil. The purpose of the experiment was to analyze the processes responsible for release of fluorine and characteristics of high-fluorine soil created through leaching of in situ high-fluorine soil, so as to provide data to support theoretical studies of in situ treatment of SHFGW.

#### 2.3.1. Soil Column Leaching Experiment

**Figure 2:**
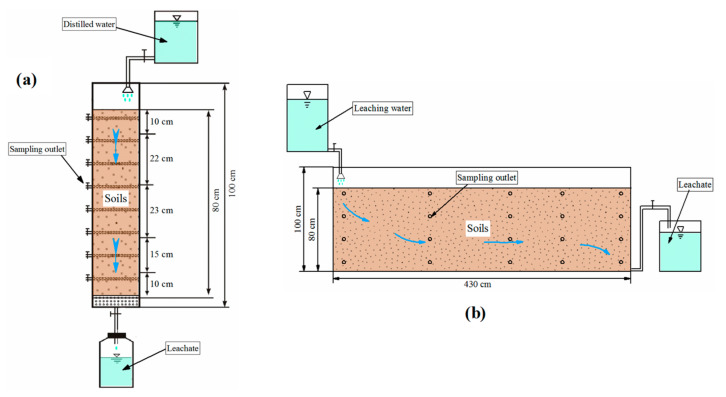
Schematic of leaching experiment. (**a**) Soil column leaching experiment, (**b**) soil tank leaching experiment.

Figure 2a shows a schematic of the soil column leaching experiment. The soil column device used was 100 cm high and 30 cm in diameter and composed of plexiglass. Soils collected from the village of Damoujia were loaded into the soil column device according to their in situ horizons and occurrences. Distilled water was concurrently injected from the bottom to saturate the soil column. At saturation of the soil column, water samples were collected from the sampling outlets, and the switch to activate the leaching of distilled water at the top of the soil column and the outlet at the bottom were activated to initiate the experiment. Water samples were collected from the sampling outlets every 24 h and fluoride concentrations of the samples were measured. The experiment lasted 1046 h and used a total of 300 L of distilled water. Water sampling occurred over a total of 44 sampling periods and 391 water samples were collected. At the end of the experiment, the water-soluble fluoride contents of soils in different layers of the soil column were measured and compared with those of soils before the experiment. During the experiment, the fluoride concentration and pH of distilled water were 0.01 mg·L^−1^ and 7.14, respectively.

#### 2.3.2. Soil Tank Leaching Experiment

Figure 2b shows a schematic of the soil tank leaching experiment. Soils collected from the village of Damoujia were loaded into the soil tank device according to their in situ horizons and occurrences. Tap water was concurrently injected from the bottom to saturate the soil tank. At saturation of the soil tank, water samples were collected from the sampling outlets and the switch to initiate leaching on the upper left-hand side and on the right-hand side of the soil tank was activated to initiate the experiment. Water samples were collected from the sampling outlets every 24 h and the fluoride concentration of each sample was measured. Tap water was used in this experiment due to the larger water requirement. The fluoride^−^ concentration of tap water ranged from 0.15 mg·L^−1^ to 0.28 mg·L^−1^, similar to that of rainwater in the study area [56], whereas pH ranged from 7.30 to 7.45. The experiment lasted 2982 h, and water sampling was performed over 46 periods with a total of 718 water samples collected.

### 2.4. Chemical Analyses

Water samples collected during the field investigation were analyzed for pH, total dissolved solids (TDS), K^+^, Na^+^, Ca^2+^, Mg^2+^, Cl^−^, SO_4_^2−^, HCO_3_^−^, CO_3_^2−^ and F^−^. TDS and pH were determined in the field using a portable water quality multi-meter (Hash Qh40d), whereas K^+^, Na^+^, Ca^2+^ and Mg^2+^ were determined by a TAS-990 atomic absorption spectrometer, SO_4_^2−^ by a TU-1901 spectrophotometer, Cl^−^, HCO_3_^−^ and CO_3_^2−^ by acid-base titration and F^−^ by the fluoride ion selective electrode method. Soil samples collected from Nanxiegou and Zhoujiazhuang were analyzed for total fluoride and water-soluble fluoride contents and soil samples from Damoujia and at the end of the experiment were analyzed for water-soluble fluoride content only. Total fluoride content was determined by the alkali-fusion method [58], and the water-soluble fluoride content was indirectly determined by the F^−^ concentration of the leached solution of soil and deionized water soaked for 24 h at a solid-liquid ratio of 1:5. F^−^ concentrations of water samples collected from the soil column leaching experiment and soil tank leaching experiment were determined by the fluoride ion selective electrode method. Parallel samples, blank samples and standard samples were also analyzed for quality control, with the relative errors of all samples being within 5%.

## 3. Results and Discussion

Data of the results of the chemical analysis of water samples collected from the field investigation, the soil column leaching experiment and the soil tank leaching experiment are show in the Supplementary File.

### 3.1. Analysis of the Processes Responsible for the Formation of Shallow High-Fluorine Groundwater in the Jiaolai Basin

#### 3.1.1. Sources of Fluoride in Groundwater

Three main sources of fluoride in groundwater exist, namely, fluoride in the (1) atmosphere, (2) endogenous groundwater and (3) rocks and soils [7]. Although rainwater F^−^ concentrations in the study area range from 0.2 mg·L^−1^ to 0.4 mg·L^−1^, there are no active volcanoes in the study area and surrounding areas. Data collected during the 1970s showed that there has been no change in F^−^ concentration of groundwater between that time and now. Therefore, fluoride in the atmosphere can be discounted as a main source of fluoride in groundwater in the study area. There is no endogenous water in shallow groundwater. In addition, high-fluoride aquifers in the study area are mainly shallow loose sediment aquifers in the low-lying areas on both sides of the Jiaolai River, and there is no endogenous water in the deep bedrock aquifers in the surrounding groundwater recharge area. Therefore, endogenous groundwater can be discounted as a source of fluoride [49].

The underlying bedrock strata found in the Jiaolai Basin are sandstone, conglomerate, mudstone, volcanic rock and pyroclastic rocks of the Mesozoic Cretaceous period. Although all rocks of the Mesozoic strata in the study area contain fluoride, there are differences in fluoride-bearing minerals and fluoride content among the different rocks due to the different formation times and lithology. The results of analysis of the total and soluble fluoride content of rocks and soils in the study area shown in Table 3 indicate that fluoride in rocks and soils is likely the main source of fluoride in groundwater in the Jiaolai Basin.

#### 3.1.2. Factors Influencing Fluoride Enrichment

The enrichment of groundwater fluoride is a geochemical process accompanied by the evolution of the chemical composition of water. The fluoride content of rocks and soils cannot simply be used to determine the amount of fluoride entering groundwater as the combined action of many factors is responsible for the migration and enrichment of fluoride in groundwater, such as geological background, climate, topography and hydrogeology, particularly the hydrogeochemical environment.

##### Climate

**Figure 3:**
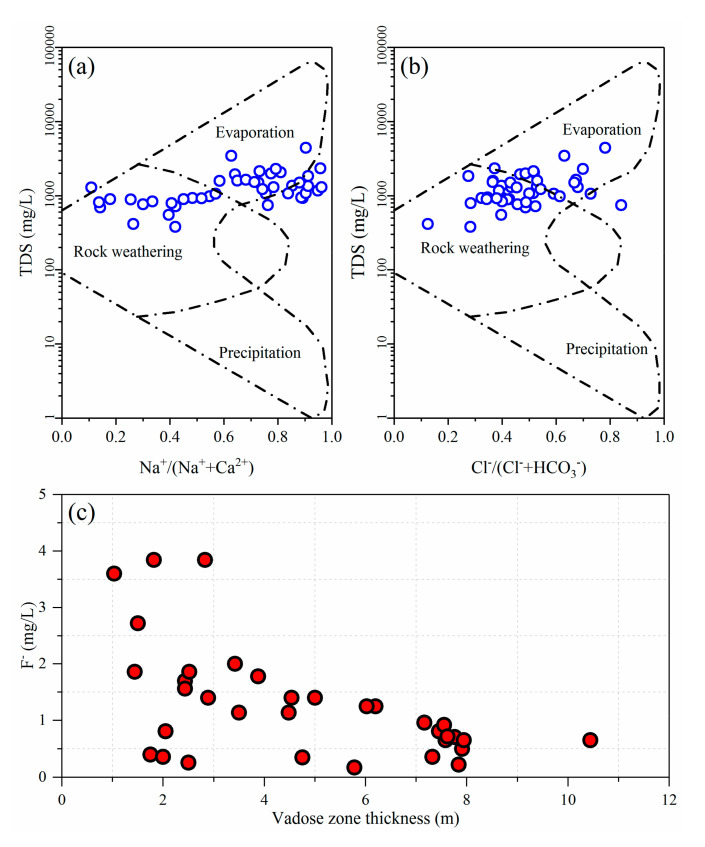
Influence of evaporation on fluoride in the groundwater of the Jiaolai Basin. (**a**) and (**b**) are the Gibbs diagram of groundwater, (**c**) is the relationship between F^-^ and vadose zone thickness.

The precipitation and evaporation characteristics of the regional climate have an important effect on the water quantity and water chemistry of shallow groundwater. The Jiaolai Basin falls into a typical semi-humid monsoon climate zone characterized by relatively low precipitation, high evaporation and an uneven distribution of rainfall and evaporation during the year. Groundwater loss in the Jiaolai Basin mainly occurs through evaporation, and high evaporation will promote the concentration and enrichment of chemical components in groundwater [63]. As shown in Figure 3a,b, the Gibbs analysis of groundwater in the Jiaolai Basin indicates that the formation of groundwater chemical components in the Jiaolai Basin is dominated by evaporation and rock weathering reactions. Analysis of F^−^ concentration and vadose zone thickness showed that high F^−^ concentrations mainly occurs in shallow buried groundwater (Figure 3c). These results indicate that evaporation plays an important role in the migration and enrichment of fluoride in shallow groundwater.

##### Topography

**Figure 4:**
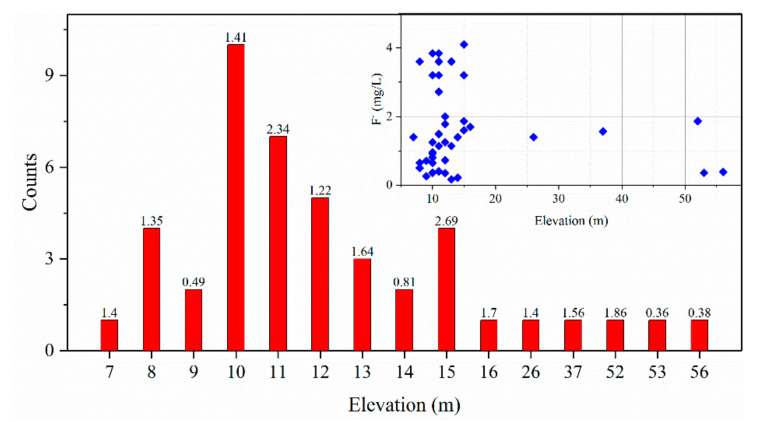
Influence of topography on fluoride in groundwater of the Jiaolai Basin, Jiaodong Peninsula, eastern China. The data label above the bar is the average F^−^ concentration of all water samples at this elevation.

The Jiaolai Basin is surrounded by low and sloping hills, with Jiaolai alluvial plain in the center. The topography of the basin inclines from the surrounds to the center, thereby providing a condition that promotes the concentration of fluoride in groundwater [53]. Figure 4 shows the relationship between groundwater F^−^ concentration and elevation. Water samples with high F^−^ concentration were mainly concentrated in the lower elevation area, with the F^−^ concentrations of groundwater tending to increase with decreasing of elevation. These results indicated that fluoride shows the same pattern of convergence as surface water and groundwater, moving from high to low elevations.

##### Groundwater Flow Dynamics

**Figure 5:**
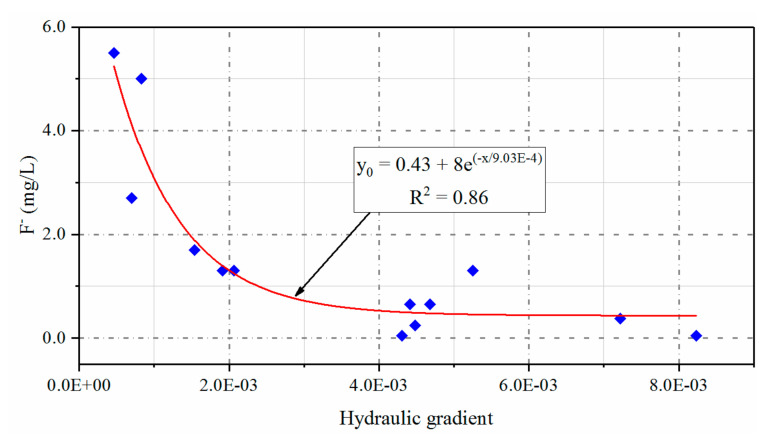
Influence of hydraulic gradient on fluorine in groundwater of the Jiaolai Basin, Jiaodong Peninsula, eastern China.

The hydrodynamic conditions resulting from hydrogeological conditions affects fluoride enrichment in groundwater. Typically, the more well-developed the hydrodynamic processes affecting groundwater, the lower the fluoride content [64]. Within the area comprising the Jiaolai Basin, Jiaodong Group and Fenzishan Group of the Archean, Proterozoic geology is exposed in the northeast, whereas the Qingshan and Wang’s groups of the Cretaceous period are exposed in the west and south. The interior of the basin is covered with Quaternary alluvial strata with a lithology comprising mainly of clay and sub-clay with slow groundwater flow. These characteristics provide beneficial hydrodynamic conditions for the enrichment of fluoride in groundwater [53,55,65]. Cui [66] conducted a statistical analysis of the relationship between groundwater hydraulic gradient and F^−^ concentration in the Jiaolai Basin based on data collected by the Shandong Geological Survey. The results of the Cui [66] study showed that the F^−^ concentration of groundwater was negatively correlated with hydraulic gradient, and the F^−^ concentration of groundwater increased rapidly with a decrease in hydraulic gradient. Figure 5 shows a scatter diagram of the relationship between groundwater hydraulic gradient and F^−^ concentration based on the previous survey data for the study area (Figure 5). The results shown in Figure 5 are basically identical to those of Cui [66], where there is a rapid drop in the groundwater F^−^ concentration in the study area with an increase in the hydraulic gradient. This result indicates that more well-developed hydrodynamic conditions effectively restrict the enrichment of fluoride in groundwater, whereas less well-developed hydrodynamic conditions with slow groundwater flow are more conducive to the enrichment of groundwater fluoride. Less well-developed hydrodynamic conditions and slow groundwater flow will increase the retention time of the fluoride-containing groundwater. Furthermore, the duration of the action of evaporation on groundwater will increase, leading to an increase in the concentration of fluoride in groundwater. Conversely, fast groundwater flow will result in the rapid transport of fluoride in groundwater, thereby reducing the influence of evaporation on groundwater fluoride. This phenomenon is shown in the study area, from the clay sedimentary area with less well-developed hydrodynamic conditions to the sand sedimentary area near the Jiaolai River with more well-developed hydrodynamic conditions in which there is a gradual reduction in groundwater F^−^ concentration (Figure 1c).

##### Hydrochemistry

**Figure 6:**
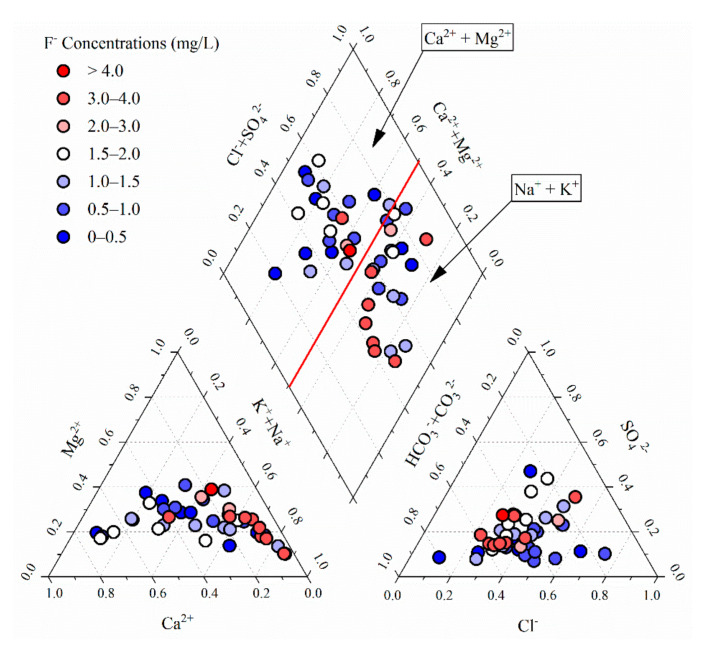
Influence of hydrochemical conditions on fluoride in groundwater of the Jiaolai Basin.

Fluoride enrichment in groundwater requires the presence of certain hydrochemical conditions [67]. Fluoride content in water is controlled by the solubility of fluorite, which is dependent on the presence of Ca in water [2]. The hydrochemical type of HFGW is generally Na-rich/Ca-poor water [2,9]. As shown in Figure 6, the present study analyzed the relationship between F^−^ concentration and hydrochemistry of groundwater in the Jiaolai Basin. From the results, it is evident that groundwater in the Jiaolai Basin with a high F^−^ concentration also showed a high and low Na^+^ and Ca^2+^ equivalent fraction, respectively. This phenomenon can be explained by the following reactions:

(1) $${\mathrm{CaF}}_{2}{=\mathrm{Ca}}^{2+}{+2F}^{-}$$

(2) $${\mathrm{CaF}}_{2}{+2\mathrm{NaHCO}}_{3}{=\mathrm{CaCO}}_{3}{+2\mathrm{Na}}^{+}{+2F}^{-}{+H}_{2}{O+\mathrm{CO}}_{2}$$

**Figure 7:**
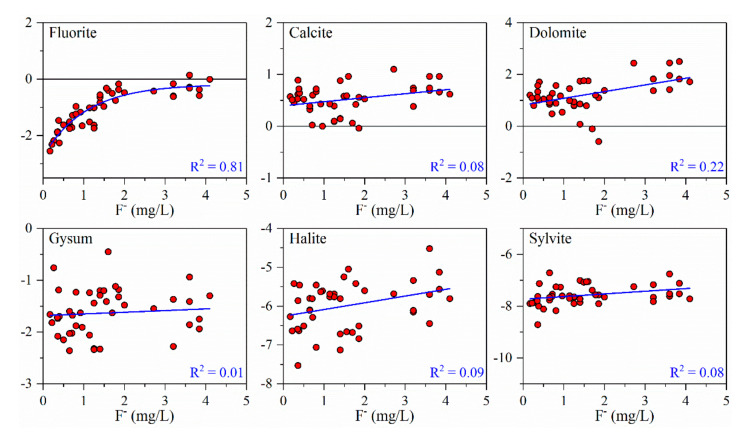
Saturation indices of the minerals in groundwater of the Jiaolai Basin, Jiaodong Peninsula, eastern China.

The present study calculated the saturation indices of several common minerals such as fluorite, calcite and dolomite in each water sample using the measured water sample chemistry data, and as shown in Figure 7, the relationship between the saturation index of each mineral and the F^−^ concentration in water samples was analyzed. A positive or negative saturation index indicates the supersaturated or unsaturated state of minerals in groundwater, respectively. As shown in Figure 7, all saturation indices of fluorite, gypsum, halite and sylvite were negative, whereas those of calcite and dolomite were positive. Moreover, the saturation indices of all minerals increased with the increasing F^−^ concentration. In particular, fluorite showed a high positive correlation with F^−^ concentration. These results indicated that fluorite, gypsum, halite and sylvite in water samples gradually transformed from an unsaturated to saturated state with increasing F^−^ concentration, whereas the degree of supersaturation of calcite and dolomite gradually increased. The saturation indices of Ca-containing minerals such as fluorite, calcite and dolomite increased with the increasing F^−^ concentration, indicating that the presence of a large amount of Ca^2+^ in the water sample was not conducive to further increase in F^−^ concentration. Therefore, the hydrochemical type of high Na^+^ and low Ca^2+^ in the Jiaolai Basin provides favorable conditions for the enrichment of F^−^ in groundwater.

#### 3.1.3. Model to Represent the Formation of SHFGW in the Jiaolai Basin

Many factors contribute to the formation of HFGW in the Jiaolai Basin, including stratigraphic lithology, topography, hydrogeology, hydrochemistry and climate.

**Figure 8:**
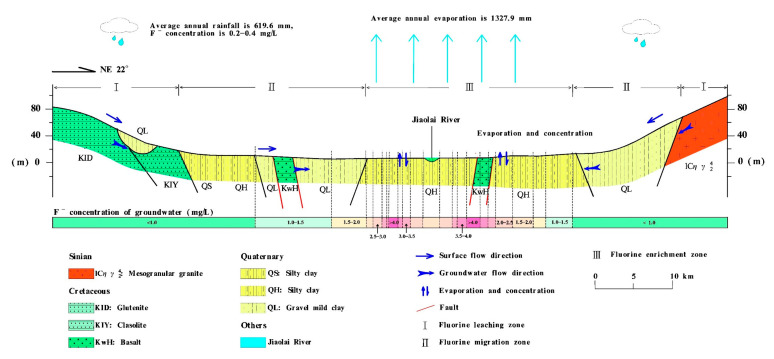
Model explaining the formation of shallow high-fluoride groundwater in the Jiaolai Basin, Jiaodong Peninsula, eastern China.

Sedimentary strata of the Laiyang, Qingshan and Wang’s groups of the Cretaceous period situated around the Jiaolai Basin and strata deposited in the Quaternary layer of the basin are rich in fluoride, which can provide a stable source of fluoride for groundwater fluoride enrichment during the long-term geological process. The Jiaolai Basin is surrounded by low and sloping hills, with Jiaolai alluvial plain in the center. Surface water and groundwater flow from the hilly areas on both the south and north sides of the alluvial plain in the center of the basin converge in the low-lying areas on both sides of the Jiaolai River. Since the Quaternary sediments in the center of the basin are composed mainly of both fine clay and sub-clay and since old strata block the entrance of Jiaozhou Bay in the South and Laizhou Bay in the north, groundwater in the study area is shallow with slow runoff. At the same time, fluoride in the old strata surrounding the basin has dissolved into the groundwater and has accumulated in the center of the basin along with runoff, which has further increased the fluoride content of the groundwater environment. Moreover, high evaporation and low rainfall climatic characteristics of the Jiaolai Basin facilitate conditions under which evaporation is the main process contributing to groundwater loss. Long-term evaporation has promoted further concentration and enrichment of fluoride in shallow groundwater, resulting in an increase in groundwater F^−^ concentration and finally resulting in the formation of SHFGW areas in the center of Jiaolai Basin (Figure 8).

### 3.2. In Situ Treatment of SHFGW

#### 3.2.1. Introduction of In Situ Treatment

Gao et al. [49] proposed the concept of *in Situ Fluoride Dispelling* through field investigation as a method of reducing the hazard to human health resulting from the high fluorine environment in the Jiaolai Basin and referred to the saline-alkali land treatment method. Formation of saline-alkali land is due to either natural or artificial factors which result in an increase in the groundwater level and increase in groundwater salinity coupled with drought and an increase in evaporation, salinization or alkalization occurring in surface soils. Measures to control saline-alkali land mainly include applying specific fertilizer, planting specific crops, adding amendments and physical drainage. Physical drainage methods are most appropriate for the treatment of SHFGW in the Jiaolai Basin.

**Figure 9:**
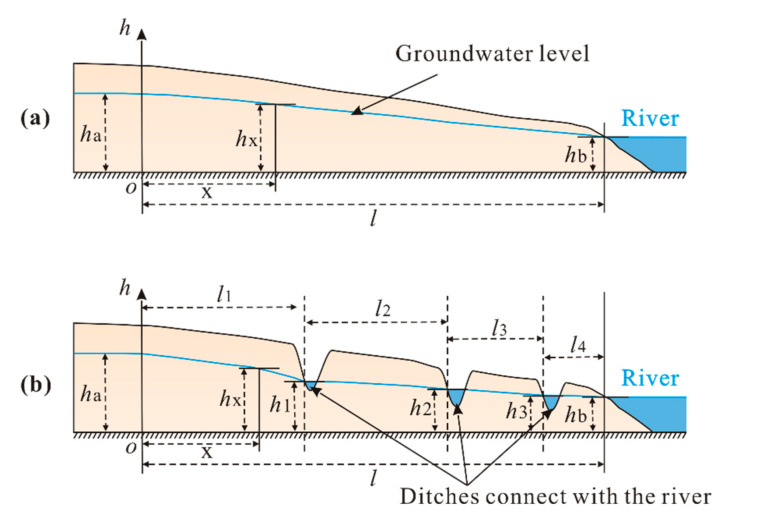
Schematic diagram of physical drainage measures. (**a**) Initial groundwater level, (**b**) the groundwater level after the physical drainage measures. *h*—is the groundwater level, *l*—is the distance between two water-passing sections, and x—is any point between two water-passing sections.

Physical drainage measures are mainly applied in areas in which there is high salinization and shallow groundwater and in which a drainage system can be established (Figure 9). In the physical drainage measures, reasonable spacing of ditches can effectively control the water level of shallow groundwater (Formula (3)) [68] and accelerate the loss of shallow groundwater.

(3) $$h_{x}^{2}=h_{a}^{2}+\frac{h_{b}^{2}-h_{a}^{2}}{l}x+\frac{W}{K}\left(lx-x^{2}\right),$$

where *W* is the amount of infiltration per unit area; *K* is the permeability coefficient.

The influence of evaporation on groundwater can be largely eliminated when the groundwater level is below 1 m [69,70]. Therefore, the water level of a drainage ditch should extend below 1 m to effectively drain the salt leached from surface soils of saline-alkali areas, thereby promoting the desalinization of soil. Since this method requires the loss of shallow groundwater through ditches, it is only suitable for high-fluoride areas with shallow groundwater. This approach is referred to a form of in situ treatment of SHFGW since it does not remove the water-bearing medium, nor does it require the extraction of HFGW for artificial defluorination [71]. In other words, this approach to in situ treatment of SHFGW uses the ditch network to accelerate the loss of shallow groundwater from the high-fluoride area through surface runoff concurrently with the leaching of low-fluoride precipitation or irrigation water through shallow high-fluoride soil, thereby reducing soil fluoride content so as to expel and reducing fluoride.

This in situ treatment method requires a change in hydrogeological conditions to achieve a reduction in groundwater F^−^ concentration in SHFGW areas. Similar to the treatment of saline-alkali land, the purpose for the excavation of ditches is to divert surface water runoff to allow the loss of SHFGW to surface runoff, resulting in the loss of fluoride and an increase in the depth of shallow groundwater. Thus, the influence of evaporation on groundwater F^−^ concentration is lowered while fluoride is lost from high-fluoride areas along with surface runoff. The approach of reducing groundwater levels by excavating ditches to drain shallow groundwater to surface runoff has already been practiced for the treatment of saline-alkali land [72,73]. However, many challenges persist in achieving the goal of in situ treatment due to the active chemical properties of fluoride, such as the characteristics of fluoride leaching from shallow high-fluoride soils and its transport in shallow water-soil systems.

#### 3.2.2. Leaching and Transport of Fluoride in a Shallow Water-Soil System

##### Distribution of Fluoride in Shallow Soils

**Figure 10:**
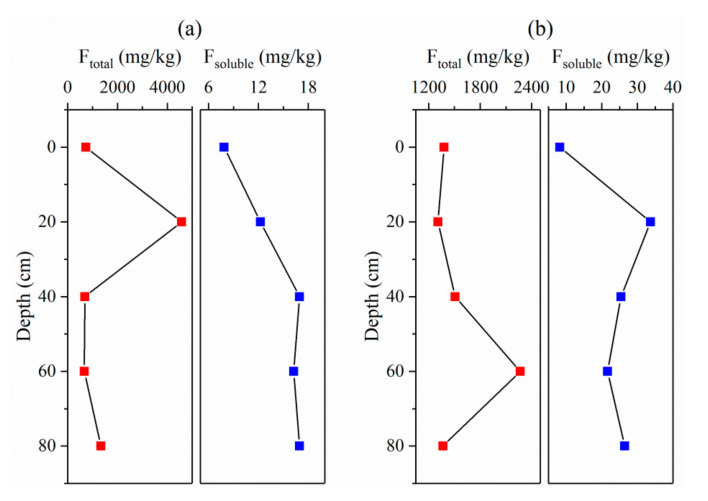
Distribution of fluoride in shallow soils in the Jiaolai Basin, Jiaodong Peninsula, eastern China. (**a**) Nanxiegou village, (**b**) Zhoujiazhuang village.

Figure 10 shows the distribution of fluoride in shallow soils at two locations in the Jiaolai Basin. Nanxiegou and Zhoujiazhuang fall within areas of relatively high and relatively low groundwater fluoride areas in the Jiaolai Basin, respectively. The highest soil total fluoride content in Nanxigou appeared at a depth of 20 cm and reached 4570 mg·kg^–1^, whereas the average soil total fluoride content in the depth range of 0 cm to 80 cm was 1600 mg·kg^–1^. The highest soil total fluoride content in Zhoujiazhuang appeared at a depth of 60 cm and reached 2270 mg·kg^–1^, averaging 1570 mg·kg^–1^. In contrast to the change in total fluoride content, the change in soil water-soluble fluoride showed similar regularity at the two locations, with average contents of water-soluble fluoride at Nanxiegou and Zhoujiazhuang being 14.1 mg·kg^–1^ and 23.1 mg·kg^–1^, respectively. The minimum water-soluble fluoride content occurred on the surface whereas the maximum occurred at a soil depth range of 20 cm–40 cm. This phenomenon indicates that shallow soils in the high-fluoride area have become a site of semi-permanent storage of water-soluble fluoride after long-term alternation of leaching and evaporation, and a seasonal dynamic balance of water-soluble fluoride between water and soil has formed [7]. A reduction in groundwater level can easily change this balance, which will lower the effect of evaporation and strengthen the leaching of water-soluble fluoride from shallow soils, thereby facilitating the dissolution of a large amount of fluoride from shallow soils to groundwater.

##### Characteristics of Fluoride Leaching and Transport in a Shallow Water-Soil System

Leaching of fluoride from soils is a dynamic process in which desorption and adsorption of fluoride between soil and groundwater occur simultaneously. An increase in the rate of desorption to a level exceeding that of adsorption would result in the leaching of soil fluoride into groundwater, whereas the converse would result in fluoride in groundwater being adsorbed by soil. The rates of desorption and adsorption are influenced by fluoride content in soil and groundwater as well as hydrogeochemistry conditions [74].

**Figure 11:**
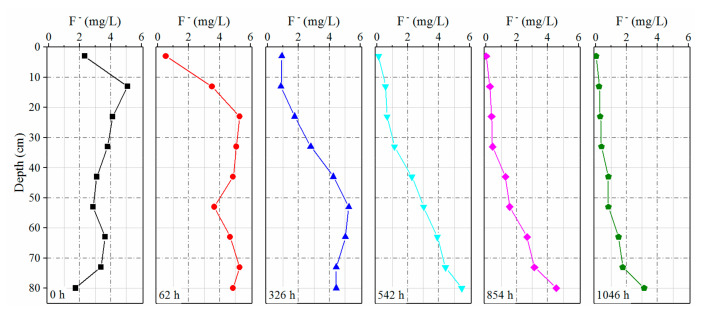
F^−^ concentration of the soil solution in the soil column of a soil column leaching experiment.

**Figure 12:**
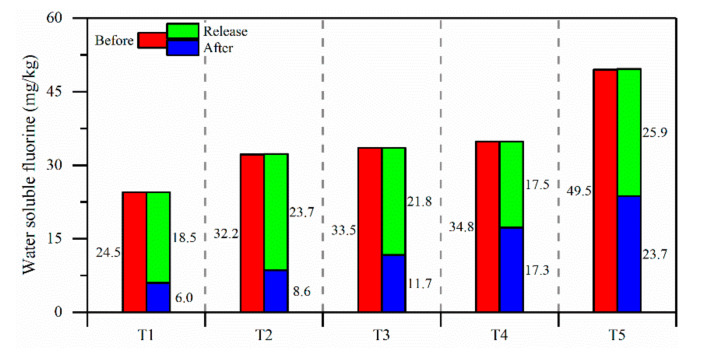
Change of water-soluble fluoride of soils before and after a soil column leaching experiment.

*Soil column leaching experiment.*Figure 11 shows the results of the soil column leaching experiment. During the experiment, the upper soil of the soil column was leached directly by distilled water with a lower F^−^ concentration, and the highest rate of leaching occurred for water-soluble fluoride whereas leaching of lower soils was relatively delayed. The soil solution F^−^ concentration in the soil column showed a peak in the vertical direction and moved downward with progression of the experiment. From 0 h to 326 h during the experiment, the range of the peak shifted from 10 cm–20 cm of the soil column to 50 cm–60 cm. With the downward movement of the peak, there was a decrease in the soil solution F^−^ concentration in the upper soil column, whereas that of the lower section increased. The F^−^ concentrations of the soil solution at different depths began to show a downward trend up until the peak moved down to the bottom of the soil column. Figure 12 shows a comparison of changes in the content of water-soluble fluoride for different layers of the soil column before and after the experiment. A large amount of water-soluble fluoride in the soil column dissolved into water after a long period of leaching, and the amount of water-soluble fluoride dissolved from soils in different layers of the soil column reached to 50.4%–75.5% of total water-soluble fluoride. During the experiment, a total of 983 mg of fluoride was dissolved from soil into water, following which it was discharged from the soil column.

**Figure 13:**
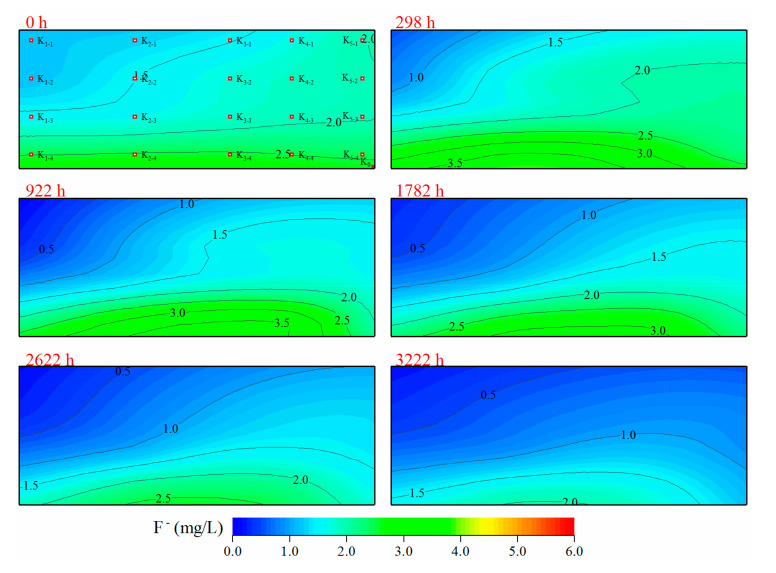
F^−^ concentration of the soil solution in a soil tank experiment over different periods.

*Soil tank leaching experiment.*Figure 13 shows the results of the soil tank leaching experiment. The distribution of F^−^ concentration in the soil tank showed a good regularity at 0 h. Higher F^−^ concentrations occurred at the lower part of the soil tank, with F^-^ concentrations decreasing at higher parts of the soil tank, forming a relatively low F^−^ concentration region in the recharge area of the upper left corner. After the start of the experiment, the low fluoride area in the upper left corner of the tank gradually expanded through the leaching of tap water, and F^−^ concentration gradually decreased with a large amount of water-soluble fluoride gradually dissolving in water. Under the stable runoff conditions in the experiment, a large amount of water-soluble fluoride was dissolved from soil, carried away by water and discharged from the soil tank, with the fluoride content in the soil tank showing a continuous decline. Leaching of fluoride from soils in areas with SHFGW is a slow process dependent on the recharge of low-fluoride water and unobstructed runoff and discharge. From the beginning to the end of the experiment, the average F^−^ concentration of soil solutions in the soil tank decreased from 2.42 mg·L^−1^ to 1.05 mg·L^−1^. In addition, a total of 5930 mg of water-soluble fluoride was dissolved from soil into water and discharged from the soil tank with runoff.

#### 3.2.3. Recommendations for In Situ Treatment of SHFGW in the Jiaolai Basin

The results of the leaching experiments indicated that groundwater recharge by low-fluoride water along with an improvement in runoff and discharge conditions can improve the outcomes of the leaching of fluoride from soils achieve better results, which is an in situ approach to the treatment of SHFGW areas. A large amount of water-soluble fluoride in the shallow high-fluoride water-soil system of the high-fluoride areas in Jiaolai Basin is dissolved into groundwater under leaching by waters with a low F^−^ concentration. The soil column leaching experiment indicated direct leaching of the upper soils of the soil column and the largest rate of leaching was obtained for water-soluble fluoride whereas leaching of lower soils was relatively delayed. A total of 983 mg of soil water-soluble fluoride in the soil column was leached into water, following which it was discharged out of the soil column, and the amount of soil water-soluble fluoride that was dissolved reached up to 50.4%–75.5% of the total by the end of the experiment. The soil tank experiment showed similar results. A total of 5930 mg of water-soluble fluoride was dissolved into water and discharged out of the soil tank with runoff. Wang et al. [75] obtained similar results in leaching experiments using eight different soil types in which 400 g–700 g of each soil type was leached with 2500 mL of low-fluorine water and the water-soluble fluorine removal rates ranged from 11% to 50.8%.

The leaching of fluoride from soils in a complete groundwater flow system incorporating recharge, runoff and discharge starts from the recharge area and proceeds along the groundwater flow path [70]. Therefore, the leaching of fluoride from soils in an SHFGW area is a gradual process which requires recharge by low-fluoride water and unobstructed runoff and discharge conditions.

The in situ treatment of an SHFGW area through the construction of a ditch network can act to strengthen the hydraulic connection between shallow groundwater and surface water, shorten the runoff path of shallow groundwater and provide beneficial runoff and discharge conditions for the leaching of fluoride in a shallow water-soil system. Under leaching by low-fluoride rainwater, water-soluble fluoride of shallow soils is able to dissolve into groundwater, and the presence of a ditch network can facilitate the discharge of groundwater flow out of the high-fluoride areas and into rivers. Moreover, the construction of a ditch network can enhance the discharge of SHFGW to surface runoff, reduce the volume of shallow groundwater and increase groundwater depth, thus lowering the influence of evaporation on the F^−^ concentration of groundwater and preventing the re-formation of SHFGW.

## 4. Conclusions

The existence of SHFGW in the Jiaolai Basin poses a risk to the health and social development of residents. The present study conducted a field investigation to identify the processes responsible for the formation of SHFGW. In addition, shallow soils taken from high-fluoride areas were used in laboratory experiments to identify the characteristics of fluoride leaching and migration in a shallow water-soil system. The results of the field investigation and experiments were used to identify possible in situ SHFGW treatment methods. The following conclusions were drawn in the present study:

(1) The formation of SHFGW in the Jiaolai Basin is as a result of many factors, including stratigraphic lithology, topography, hydrogeology, hydrochemistry and climate. The Cretaceous and Quaternary strata around and inside the basin act as the source of fluoride. The terrain of the basin with slow groundwater flow promotes the convergence of groundwater. The hydrochemical composition of the majority of groundwater in the center of the basin being of a Na-rich/Ca-poor type provides beneficial hydrodynamic and hydrochemical conditions for the formation of SHFGW. Finally, the climate conditions of the basin characterized by high evaporation and low rainfall facilitate the concentration and enrichment of fluoride in shallow groundwater.

(2) The results of the leaching experiments indicated that recharge by low-fluoride water and high runoff and discharge conditions can achieve a better outcome for the leaching of fluoride from soils. A total of 983 mg of soil water-soluble fluoride the soil column was leached into water, following which it was discharged out of the soil column, with the amount of soil water-soluble fluoride discharged reaching 50.4%–75.5% of the total amount at the end of the experiment. The soil tank experiment showed similar results. A total of 5930 mg of water-soluble fluoride was dissolved into water and discharged out of the soil tank with runoff.

(3) In situ treatment of SHFGW may be an effective measure to mitigate the problem of SHFGW in the Jiaolai Basin. The construction of a ditch network in the SHFGW area can strengthen the hydraulic connection between shallow groundwater and surface water, provide beneficial runoff and discharge conditions for the leaching of fluoride in a shallow water-soil system and facilitate the dissolution of water-soluble fluoride of shallow soils into groundwater and the movement of SHFGW out of the high-fluoride areas through river outflow. Moreover, the construction of a ditch network could reduce the level of shallow groundwater and increases groundwater depth, thus lowering the influence of evaporation on groundwater F^−^ concentration and preventing the re-formation of SHFGW.

## Figures and Tables

**Table 1:** A summary of recent studies on the distribution, source and formation of high-fluoride groundwater.

References	Area	Main Conclusions
Martínez et al. [24]	Quequen River Basin in the Argentine Pampa	Samples with fluoride contents between 0 mg L^−1^ and 3 mg L^−1^ were widely distributed in the catchment and samples with fluoride contents between 3 mg L^−1^ and 6 mg L^−1^ corresponding to a smaller area in the central–west border.
Vasil’chuk et al. [25]	Minusinsk Basin	The fluoride content in the soils and plants near the Sayanogorsk aluminum smelter reached maximum concentrations of 10 ppm–12 ppm.
Liu et al. [26]	Manas River Basin, Northwest China	Fluoride^−^ concentrations progressively increased with increasing residence time and increasing well depth northwest of the alluvial–fluvial plain, and vertical leaching by irrigation return flow and mixing with pore water were identified as the dominant processes driving the migration of fluoride^−^ in the groundwater flow system.
Adimalla and Venkatayogi [27]	Medak district, South India	The fluoride concentration in groundwater ranged between 0.23 mg L^−1^–7.4 3 mg L^−1^ with an average concentration of 2.7 3 mg L^−1^, and the characteristics of the groundwater flow regime, long residence time and the extent of groundwater interaction with rocks were found to be the major factors influencing the concentration of fluoride.
Gray [28]	New Zealand	The total fluoride concentrations in topsoil (0 cm–7.5 cm) that received 188 kg ha^−1^ and 376 kg ha^−1^ single superphosphate (SSP) fertilizer increased from 251 mg·kg^−1^ to 349 mg·kg^−1^ and 430 mg·kg^−1^, respectively. The rates of fluoride accumulation were estimated at 1.1 kg·ha^−1^·yr^−1^ and 2.1 kg·ha^−1^·yr^−1^, respectively.
Li et al. [29]	Cuvelai-Etosha Basin and Kaokoveld region, northwestern Namibia	The salinity and concentrations of As, U and F^−^ in some groundwater samples exceeded of the World Health Organization (WHO) standards and the low Ca^2+^ concentrations in groundwater and sediments rich in fluoride favored the formation of high-fluoride groundwater.
Chen et al. [30]	Jiahe estuarine region, China	Investigated the geo-chemical processes occurring during mixing of fresh water and seawater and found that estuarine water had high levels of fluoride due to the seawater mixing.
Berger et al. [31]	Laxemar, southeast Sweden	Proposed two mechanisms explaining the overall high fluoride levels in fracture groundwater, namely, weathering/dissolution of fluoride-rich minerals and long water residence times, which favor water-rock interaction and the build-up of high concentrations of dissolved fluoride.

**Table 2:** The main stratum and lithology features of the Jiaolai Basin, Jiaodong Peninsula, eastern China [53,54,55].

Period	Group	Formation	Distribution	Features
Quaternary		Qhy	Distributed along rivers	Mainly for gravel and coarse sand deposits, forming riverbed and low river floodplain
		Qhl	Distributed along the sides of rivers	Mainly silt and sandy clay, forming high floodplain
		Qhh	Widely developed inside the basin	Mainly sandy clay and silty clay, partially containing gravel and iron-manganese nodules
		Qpd	Mainly distributed near Gaomi County and with sporadic distributions in the southeast	Mainly well sorted silty clay or sandy clay, often with several layers of irregular gravel
Cretaceous	Wang’s Group	K_1_h	Mainly hidden under the Quaternary	Mainly fine sandstone and siltstone
	Qingshan Group	K_1_b	Mainly distributed in the eastern part of the study area, sporadic in the south and west	Mainly Anshan mass block breccia sandwiched tuff, lithic tuff
	Laiyang Group	K_1_q	Mainly distributed in the southern part of the study area, partially hidden under the Quaternary	Mainly siltstone and fine feldspathic sandstone
		K_1_d		Mainly conglomerate, with gravelly coarse sandstone and fine siltstone, siltstone
		K_1_y		Mainly medium-grained feldspar sandstone with pebbled sandstone, medium-fine feldspar sandstone and siltstone
		K_1__ẑ_		Mainly thick-medium thick feldspar sandstone with thick layer of conglomerate and siltstone

**Table 3:** Fluoride content of rocks and soils in the Jiaolai Basin, Jiaodong Peninsula, eastern China.

Stratum	Lithology	Water-Soluble Fluoride (mg·kg^−1^)	Total Fluoride (mg·kg^−1^)	References
Quaternary	Clay	35.3	628	Meng et al. [23], Zhang et al. [59]
	Sand	26.7	466	Meng et al. [23], Zhang et al. [59], Duan [60]
	Calcareous cores	15.4	560	Zhang et al. [59], Wang et al. [61]
	Silty clay	26.8	1139	Meng et al. [23], Duan [60]
Wang’s Group	Conglomerate	20.0	600	Gao et al. [49]
	Sandstone	19.6	540	
	Mudstone	16.8	620	Meng et al. [23], Gao et al. [49]
Qingshan Group	Pyroclastic rocks	8.0	490	Gao et al. [49]
	Lava	5.8	440	
	Bentonite	10.7	1105	
	Conglomerate	5.0	390	Zhang et al. [59]
	Sandstone	11.2	650	
Laiyang Group	Sandstone	4.7	370	Gao et al. [49], Zhang et al. [59], Li et al. [62]
	Siltstone	5.0	390	
	Shale	9.5	740	

## Supplementary Materials

The following are available online at https://www.mdpi.com/1660-4601/17/21/8075/s1.

## References

[1] Xiao J., Wang L.Q., Deng L., Jin Z.D. (2019). Characteristics, sources, water quality and health risk assessment of trace elements in river water and well water in the Chinese Loess Plateau. Sci. Total Environ..

[2] Fuge R. (2019). Fluorine in the environment, a review of its sources and geochemistry. Appl. Geochem..

[3] Apambire W.B., Boyle D.R., Michel F.A. (1997). Geochemistry, genesis, and health implications of fluoriferous groundwaters in the upper regions of Ghana. Environ. Earth Sci..

[4] Pokras M. (2005). Essentials of Medical Geology: Impacts of the Natural Environment on Public Health. Environ. Heal. Perspect..

[5] Fordyce F. (2011). Fluorine: Human Health Risks. Encycl. Environ. Health.

[6] Edmunds W.M., Smedley P.L. (2013). Fluoride in Natural Waters.

[7] Adak M.K., Chakraborty S., Sen S., Dhak D. (2017). Synthesis and optimization of low-cost and high efficient zirconium-aluminium modifiediron oxide nano adsorbent for fluoride removal from drinking water. Adv. Mater. Proc..

[8] Wei W., Pang S., Sun D. (2019). The pathogenesis of endemic fluorosis: Research progress in the last 5 years. J. Cell. Mol. Med..

[9] Ali S., Thakur S.K., Sarkar A., Shekhar S. (2016). Worldwide contamination of water by fluoride. Environ. Chem. Lett..

[10] Adimalla N., Li P. (2018). Occurrence, health risks, and geochemical mechanisms of fluoride and nitrate in groundwater of the rock-dominant semi-arid region, Telangana State, India. Hum. Ecol. Risk Assess. Int. J..

[11] Narsimha A., Qian H. (2019). Hydrogeochemistry and fluoride contamination in the hard rock terrain of central Telangana, India: Analyses of its spatial distribution and health risk. SN Appl. Sci..

[12] Sariñana-Ruiz Y.A., Vazquez-Arenas J., Sosa-Rodríguez F.S., Labastida I., Armienta M.A., Aragón-Piña A., Escobedo-Bretado M.A., González-Valdez L.S., Ponce-Peña P., Ramírez-Aldaba H. (2017). Assessment of arsenic and fluorine in surface soil to determine environmental and health risk factors in the Comarca Lagunera, Mexico. Chemosphere.

[13] Li P., Oyang X., Zhao Y., Tu T., Tian X., Li L., Zhao Y., Li J., Xiao Z. (2019). Occurrence of perfluorinated compounds in agricultural environment, vegetables, and fruits in regions influenced by a fluorine-chemical industrial park in China. Chemosphere.

[14] Pan Z., Liu X., Meng W., Li C., He S., Yan C., Wang F. (2018). Geochemical characteristics of fluorine in soils and its environmental quality in central district of Guiyang. Res. Environ. Sci..

[15] Štepec D., Tavčar G., Ponikvar-Svet M. (2019). Fluorine in vegetation due to an uncontrolled release of gaseous fluorides from a glassworks: A case study of measurement uncertainty, dispersion pattern and compliance with regulation. Environ. Pollut..

[16] Vithanage M., Bhattacharya P. (2015). Fluoride in the environment: Sources, distribution and defluoridation. Environ. Chem. Lett..

[17] Sorlini S., Collivignarelli M.C., Miino M.C. (2019). Technologies for the control of emerging contaminants in drinking water treatment plants. Environ. Eng. Manag. J..

[18] Lim X. (2019). The Fluorine Detectives. Nature.

[19] Long X.T., Zhang K.N., Yuan R.Q., Zhang L., Liu Z.L. (2019). Hydrogeochemical and Isotopic Constraints on the Pattern of a Deep Circulation Groundwater Flow System. Energies.

[20] Singh P., Asthana H., Rena V., Kumar P., Kushawaha J., Mukherjee S. (2018). Hydrogeochemical processes controlling fluoride enrichment within alluvial and hard rock aquifers in a part of a semi-arid region of Northern India. Environ. Earth Sci..

[21] Mondal D., Gupta S. (2014). Fluoride hydrogeochemistry in alluvial aquifer: An implication to chemical weathering and ion-exchange phenomena. Environ. Earth Sci..

[22] Chen Q., Wei J., Wang H., Shi L., Gao Z., Liu S., Ning F., Jia C., Ji Y., Dong F. (2019). Discussion on the Fluorosis in Seawater-Intrusion Areas Along Coastal Zones in Laizhou Bay and Other Parts of China. Int. J. Environ. Res..

[23] Meng C., Wang C., Ma Z., Shang Q., Zheng X. (2018). Distribution and impact factors of high fluoride groundwater in Pingdu City. Environ. Sci. Technol..

[24] Martínez D.E., Londoño O.M.Q., Massone H.E., Buitrago P.P., Lima L. (2011). Hydrogeochemistry of fluoride in the Quequen river basin: Natural pollutants distribution in the argentine pampa. Environ. Earth Sci..

[25] Vasil’chuk J.Y., Ivanova E.A., Krechetov P.P., Terskaya E.V. (2019). Heavy Metals and Fluorine in Soils and Plants of the Minusinsk Basin. Urbanization: Challenge and Opportunity for Soil Functions and Ecosystem Services.

[26] Liu Y., Jin M., Ma B., Wang J. (2018). Distribution and migration mechanism of fluoride in groundwater in the Manas River Basin, Northwest China. Hydrogeol. J..

[27] Adimalla N., Venkatayogi S. (2016). Mechanism of fluoride enrichment in groundwater of hard rock aquifers in Medak, Telangana State, South India. Environ. Earth Sci..

[28] Gray C.W. (2018). Fluorine in soils under pasture following long-term application of phosphate fertiliser in New Zealand. Geoderma Reg..

[29] Li Z., Wang G., Wang X., Wan L., Shi Z., Wanke H., Uugulu S., Uahengo C.-I. (2018). Groundwater quality and associated hydrogeochemical processes in Northwest Namibia. J. Geochem. Explor..

[30] Chen Q., Hao D.C., Wei J.C., Jia C.P., Wang H.M., Shi L.Q., Liu S.L., Ning F.Z., Ji Y.H., Dong F.Y. (2019). Geo-chemical processes during the mixing of seawater and fresh water in estuarine regions and their effect on water fluorine levels. Mausam.

[31] Berger T., Mathurin F.A., Drake H., Åström M.E. (2016). Fluoride abundance and controls in fresh groundwater in Quaternary deposits and bedrock fractures in an area with fluorine-rich granitoid rocks. Sci. Total. Environ..

[32] Zeng G., Ling B., Li Z., Luo S., Sui X., Guan Q. (2019). Fluorine removal and calcium fluoride recovery from rare-earth smelting wastewater using fluidized bed crystallization process. J. Hazard. Mater..

[33] Zhou M., Zhu S., Yi Y., Zhang T. (2016). An electrokinetic/activated alumina permeable reactive barrier-system for the treatment of fluorine-contaminated soil. Clean Technol. Environ. Policy.

[34] Sharma A., Ameta R., Benjamin S., Soni D., Sharma S., Tak P. (2017). Removal of fluoride from ground water by using bio-adsorbent like lantana camera (Jamri). Int. J. Sci. Res..

[35] Mukherjee S., Dutta S., Ray S., Halder G. (2018). A comparative study on defluoridation capabilities of biosorbents: Isotherm, kinetics, thermodynamics, cost estimation, and eco-toxicological study. Environ. Sci. Pollut. Res..

[36] Collivignarelli M.C., Abbà A., Miino M.C., Torretta V., Rada E.C., Caccamo F.M., Sorlini S. (2020). Adsorption of Fluorides in Drinking Water by Palm Residues. Sustainability.

[37] Zhang Y., Huang K. (2019). Grape pomace as a biosorbent for fluoride removal from groundwater. RSC Adv..

[38] Suneetha M., Sundar B.S., Ravindhranath K. (2015). Removal of fluoride from polluted waters using active carbon derived from barks of Vitex negundo plant. J. Anal. Sci. Technol..

[39] Arfin T., Waghmare S. (2015). Fluoride removal from water by various techniques: Review. Int. J. Innov. Sci. Eng. Technol..

[40] Kawakami T., Nishino M., Imai Y., Miyazaki H., Amarasooriya A.A.G.D. (2018). De-fluoridation of drinking water by co-precipitation with magnesium hydroxide in electrolysis. Cogent. Eng..

[41] Singh G., Kumari B., Sinam G., Kriti, Kumar N., Mallick S. (2018). Fluoride distribution and contamination in the water, soil and plants continuum and its remedial technologies, an Indian perspective—A review. Environ. Pollut..

[42] Chaudhary K., Khan S. (2016). Physiochemical Characterization of Fluoride (F) Contaminated Soil and its Microbe-Assisted Bioremediation by *Prosopis juliflora*. Plant Biol. Soil Health.

[43] Chaudhary K., Saraswat P.K., Khan S. (2019). Improvement in fluoride remediation technology using GIS based mapping of fluoride contaminated groundwater and microbe assisted phytoremediation. Ecotoxicol. Environ. Saf..

[44] Choudhary S., Rani M., Singh R., Prasad S., Patra A., Ogireddy S.D. (2019). Impact of fluoride on agriculture: A review on it’s sources, toxicity in plants and mitigation strategies. Int. J. Chem. Study.

[45] Mukherjee S., Yadav V., Mondal M., Banerjee S., Halder G. (2015). Characterization of a fluoride-resistant bacterium Acinetobacter sp. RH5 towards assessment of its water defluoridation capability. Appl. Water Sci..

[46] Carrillo-Rivera J., Cardona A., Edmunds W. (2002). Use of abstraction regime and knowledge of hydrogeological conditions to control high-fluoride concentration in abstracted groundwater: San Luis Potosí basin, Mexico. J. Hydrol..

[47] Mohapatra M., Anand S., Mishra B., Giles D.E., Singh P. (2009). Review of fluoride removal from drinking water. J. Environ. Manag..

[48] Majumdar K.K. (2011). Health impact of supplying safe drinking water containing fluoride below permissible level on flourosis patients in a fluoride-endemic rural area of West Bengal. Indian J. Public Health.

[49] Gao Z., Zhang F., An Y., Feng J., Wang M., Han K. (2014). The formation and model of highly-concentrated fluoride groundwater and *in-situ* fluoride dispelling assumption in Gaomi city of Shandong province. Earth Sci. Front..

[50] Yun Z., Gao J., Yin Y., Bian J., Chen P., Zhang B. (2014). Analysis of the monitoring results of endemic fluorosis in Shandong Province in 2011. Chin. J. Endemiol..

[51] Li J., Zhang Y., Liu Z., Ren F., Yuan J. (2007). Sedimentary-subsidence history and tectonic evolution of the Jiaolai basin, eastern China. Geol. Chin..

[52] Feng Q., Zhang Y., Xu Z., Tian F., Yang B., Zhang Y. (2018). Geochemical characteristics and paleoenvironmental analysis of dark fine grained rocks of Wawukuang and Shuinan formations in Jiaolai Basin. J. Shandong Univ. Sci. Technol..

[53] Wang J., Wei H., Bi W. (2013). Relationship between endemic fluorosis and geological environment in Jiaolai Basin of Shandong province. Shandong Land Resour..

[54] Liu Y., Liu D. (2000). Major types and propecting future of gold deposits in northeast Jiaolai basin. Shandong Land Resour..

[55] Huang T. (2000). Characteristic of regional geophysical field in Jiaolai basin and its tectonic unit division. Shandong Land Resour..

[56] Zhu X. (2013). Research on High Fluoride Groundwater Forming Mechanism in Typical Areas of Shandong Province. Master’s Thesis.

[57] Xu L., Xu Z., Chang J. (2012). Research on fluorine distribution characters of groundwater and their influencing factors in Pingdu city. China Rural Water Hydropower.

[58] Wu W., Xie Z., Xu J., Liu C. (2003). Comparison of different methods of determination of total fluoride content in soil. J. Zhejiang Univ..

[59] Zhang X., Xu J., Xing B., Wang L. (2007). Research on present condition of high fluoride areas and high fluoride groundwater—Forming mechanism in Gaomi city of Shandong province. Shandong Land Resour..

[60] Duan J. (2014). Experimental Study on Fluorine Migration and Transformation in Water-Soil System. Master’s Thesis.

[61] Wang C., Pang X., Wang H., Zeng X., Hu X., Zheng W. (2011). High-F groundwater in Gaomi city—Its genetic study. Earth Environ..

[62] Li C., Yu Z., Wu Y. (2008). Hydrogeochemical characteristics of high-fluorine groundwater in the Gaomi area, Shandong, China. Geol. Bull. China.

[63] Liu G., Yang J. (2002). Relations between soil evaporation and groundwater actions. Soils.

[64] Rao N.S. (2017). Controlling factors of fluoride in groundwater in a part of South India. Arab. J. Geosci..

[65] Liu X., Wang B., Zheng B. (2014). Geochemical process of fluorine in soil. Chin. J. Geochem..

[66] Cui H. (2012). Study on Migration and Transformation of High Fluoride Groundwater in Gaomi City. Master’s Thesis.

[67] Saxena V., Ahmed S. (2003). Inferring the chemical parameters for the dissolution of fluoride in groundwater. Environ. Earth Sci..

[68] Xue Y., Wu J. (2010). Groundwater Dynamics.

[69] Zhang Y., Li X. (2019). Dynamic evolution characteristics of typical salt-controlling factors in soils before and after spring irrigation in arid area. Shaanxi Water Resour..

[70] Zhang Q., Xu P., Qian H., Yang F. (2020). Hydrogeochemistry and fluoride contamination in Jiaokou Irrigation District, Central China: Assessment based on multivariate statistical approach and human health risk. Sci. Total. Environ..

[71] Shi M. (2015). Experimental Study on In-Situ Fluorine Dispelling of Shallow High Fluoride Groundwater. Master’s Thesis.

[72] Heng T., Wang Z., Zhang J., Li W. (2019). Development of farmland drainage technology to control saline-land in Xinjiang. J. Agric. Sci. Technol..

[73] Ghumman A.R., Ghazaw Y.M., Niazi M.F., Hashmi H.N. (2010). Impact assessment of subsurface drainage on waterlogged and saline lands. Environ. Monit. Assess..

[74] Shi M., Gao Z., Feng J., Zhang H., Cui Y., Fang S., Liu J. (2019). Characteristics and effects of fluorine release from shallow high-fluoride soils. Environ. Earth Sci..

[75] Wang W., Li R., An J., Luo K., Yang L., Li H., Li Y. (2002). Adsorption and leaching of fluoride in soils of China. Chin. Acad. Sci..

