# Supplementary material for: Formation and In Situ Treatment of High Fluoride Concentrations in Shallow Groundwater of a Semi-Arid Region: Jiaolai Basin, China
Source: Int J Environ Res Public Health. 2020 Nov 2;17(21):8075. doi: 10.3390/ijerph17218075 (PMC7663115; doi:10.3390/ijerph17218075)
Supplement: Supplementary file 1 [file ijerph-17-08075-s001.pdf]

International Journal of
Environmental Research and Public Health

MDPI

Table S1. Results of the chemical analysis of water samples collected from the field investigation in 2011.

Sample No.	pH	TDS	K^+	Na^+	Ca^{2+}	Mg^{2+}	Cl^-	$SO4^{2-}$	$HCO3^-$	$CO3^{2-}$	F^-
		mg/L	mg/L	mg/L	mg/L	mg/L	mg/L	mg/L	mg/L	mg/L	mg/L
F1-1	7.76	377.61	3.28	47.96	57.64	18.1	57.47	23.43	250.96	0	1.4
F1-2	7.13	1934.9	1.03	315.64	153.4	141.7	368.22	165.28	723.06	0	2
F1-3	7.65	1832	2.17	533.08	44.77	62.43	225.61	252.85	1021.2	0	3.84
F1-4	7.66	4431.6	2.54	1184.3	110.1	190.4	1285.6	1222.3	616.21	0	3.6
F1-5	7.4	1576.4	6.44	281.4	173.9	74.96	242.64	231.88	720.57	0	1.4
F1-6	8.09	2334.7	3.02	799.4	30.88	50.25	421.43	266.42	1222.5	0	3.84
F1-7	8.42	1303.8	2.75	275.95	65.87	79.28	317.13	219.55	255.93	14.66	2.72
F1-8	7.41	1981.4	2.47	464.08	117.3	114.6	449.1	258.92	814.99	0	3.2
F1-9	7.43	927.78	2.56	125.1	116.3	60.56	170.27	86.34	434.83	0	0.72
F1-10	8.19	931.16	2.82	254.09	26.76	32.77	114.93	82.64	412.47	0	3.2
F1-11	7.72	893.32	2.78	112.76	119.4	47.45	129.83	152.94	300.65	0	3.6
F1-12	11.7	884.97	2.32	62.35	158	44.64	125.58	155.41	298.17	0	1.4
F1-13	7.38	3449.8	2.44	664.94	342.7	105.8	657.68	1091.6	660.94	0	1.6
F1-14	7.85	942.02	1.71	235.63	26.25	47.76	134.09	85.11	434.83	0	3.2
F1-15	8.05	1363	1.92	342.75	51.46	74.91	246.9	149.24	641.06	0	3.6

Table S2. Results of the chemical analysis of water samples collected from the field investigation in 2014.

Sample No.	pH	TDS	K^{+}	Na^{+}	Ca^{2+}	Mg^{2+}	Cl^{-}	$SO4^{2-}$	$HCO3^{-}$	$CO3^{2-}$	F^{-}
		mg/L	mg/L	mg/L	mg/L	mg/L	mg/L	mg/L	mg/L	mg/L	mg/L
F2-1	8	1081.2	1.56	292	27.01	47.29	244.4	145.5	394.27	0	1.14
F2-2	7.6	1172.5	0.86	370.1	17.43	31.97	220.3	121.9	597.21	0	1.25
F2-3	7.9	1304.3	1.6	409	13.94	25.36	212.3	153.8	475.45	0	1.25
F2-4	7.4	1341.1	1.35	370.9	30.93	48.35	284.4	196	434.86	0	0.96
F2-5	7.4	1220.1	1.05	273.4	82.34	53.9	248.4	154.9	359.48	0	0.65
F2-6	7.2	1587.2	2.32	274.5	129	116.5	376.6	119.7	574.01	0	0.92
F2-7	7.3	1067	1.52	270.6	45.31	54.95	236.4	96.6	405.87	0	0.71
F2-8	8.7	743.03	13.07	148.5	40.08	40.16	214.3	39.89	69.58	2.85	0.65
F2-9	7.8	1058	1.06	169.7	111.5	62.35	338.5	79.78	217.43	0	0.36
F2-10	7.7	716.15	0.73	81.76	97.59	43.59	152.2	29.83	237.72	0	0.5
F2-11	7.6	691.19	0.83	30.13	158.1	24.31	118.2	34.69	214.53	0	0.81
F2-12	8.1	1502	3.13	399.3	47.05	58.12	372.6	231	318.9	0	0.4
F2-13	7.6	1639.5	1.94	327.7	133.3	82.96	460.7	277.5	382.68	0	0.81
F2-14	7.6	2294.3	2.43	442.8	101.1	182	584.9	517.5	431.96	0	1.49
F2-15	7.8	414.81	0.88	37.14	89.74	25.1	30.05	30.52	362.38	0	0.36
F2-16	6.7	810.76	1.16	37.47	203.9	29.33	158.2	149.2	284.11	0	1.86
F2-17	7.1	1284.7	0.7	44.73	316.3	52.05	230.4	130.1	475.45	0	0.38
F2-18	7.1	897.29	7.98	54.93	218.7	40.16	164.3	89.84	542.12	0	1.56
F2-19	7.3	832.49	2.59	81.25	140.3	62.35	156.2	162	402.97	0	1.86
F2-20	7.1	552.02	5.13	71.82	95.41	26.16	112.2	85.33	292.81	0	1.7
F2-21	7.6	1061.5	1.22	257.2	72.32	47.56	278.4	227.2	330.49	0	1.25
F2-22	7.4	1519.5	1.24	274.3	95.84	127.9	246.4	345.1	736.36	0	4.1
F2-23	7.5	975.83	1.26	126	90.61	82.96	260.4	50.64	281.21	0	0.65
F2-24	7.3	2054.7	1.27	479.9	97.59	103.6	356.6	568.2	568.22	0	1.78
F2-25	7.2	1502.2	1.19	333.2	108	66.84	264.4	312.5	600.11	0	1.14
F2-26	7.4	916.43	1.18	137.4	111.5	55.75	162.3	92.26	455.15	0	0.17
F2-27	7.2	2138	0.67	508	161.6	57.86	334.5	777.7	539.23	0	0.26
F2-28	7.4	765.14	1.33	59.85	121.1	62.35	176.3	71.11	359.48	0	0.35
F2-29	7.3	789.87	1.91	89.15	113.3	58.65	104.2	60.7	452.25	0	0.22

Table S3. F^- concentrations of the water samples collected from the soil column leaching experiment.

**Time (h)**		**0**	**3**	**7**	**12**	**18**	**38**	**62**	**86**	**110**	**134**	**158**	**182**	**206**	**230**	**254**	**278**	**302**	**326**	**350**	**374**	**398**	**422**
F^- concentration (mg/L)	K1	2.32	1.97	1.89	2.63	1.67	0.9	0.53	0.15	0.26	0.18	0.1	0.1	0.2	0.1	1.2	1.2	1.1	0.9	0.5	0.5	0.6	0.79
	K2	5.08	4.88	4.68	5.3	5.08	4.68	3.51	1.2	/	/	0.64	0.9	1	0.9	0.8	1	1.2	0.9	0.6	0.5	0.5	0.66
	K3	4.14	4.14	4.14	4.49	4.49	4.68	5.3	3.81	3.04	2.79	2.08	1.9	1.7	1.7	1.5	1.8	1.9	1.8	0.9	0.7	0.8	0.75
	K4	3.81	3.81	3.66	3.97	3.97	4.31	5.08	3.81	3.17	3.3	2.91	3.5	3.3	3.2	2.5	2.8	2.8	2.8	1.6	1.6	1.2	1.36
	K5	3.1	3.23	3.23	3.37	3.37	3.97	4.88	4.14	3.75	3.45	3.17	3.5	3.5	3.8	3.5	3.8	3.9	4.3	3.5	3	2.7	3.17
	K6	2.86	3.1	3.1	3.1	3.1	3.23	3.66	3.51	3.3	3.59	3.17	3.3	3.6	3.8	3.8	4.1	4.1	5.3	4.6	3.9	3.6	4.26
	K7	3.66	4.31	4.14	5.08	4.68	4.68	4.68	3.37	2.91	3.17	2.91	3.2	3.2	3.6	3.6	3.8	3.8	5	4.6	4.3	3.9	4.26
	K8	3.37	3.66	4.14	5.99	4.68	5.08	5.3	4.31	3.59	3.17	2.91	3	3	3.3	3.3	3.5	3.5	4.4	4.6	4.4	4.1	4.63
	K9	1.74	2.32	2.05	0.94	2.42	4.31	4.88	4.14	3.59	3.3	2.91	3.2	2.9	3.2	3.3	3.3	3.3	4.4	4.3	4.4	4.1	4.44
**Time (h)**		**446**	**470**	**494**	**518**	**542**	**566**	**590**	**614**	**638**	**662**	**686**	**710**	**734**	**758**	**782**	**806**	**830**	**854**	**902**	**950**	**998**	**1046**
F^- concentration (mg/L)	K1	0.64	0.52	0.42	0.24	0.13	0.11	0.12	0.07	0.05	0.05	0.11	0.1	0.1	0.1	0.1	0.2	0.1	0	0	0.1	0.1	0.04
	K2	0.61	0.61	0.32		0.56		0.52		0.35	0.44	0.45	0.6	0.6	0.5	0.5	0.4	0.3	0.3	0.3	0.4	0.5	0.24
	K3	0.66	0.64	0.69	0.72	0.66	0.64	0.52	0.49	0.44	0.49	0.52	0.6	0.7	0.5	0.6	0.6	0.4	0.4	0.5	0.5	0.6	0.31
	K8	4.3	4.6	4.8	4.3	4.4	4.4	4.1	4.1	3.9	3.8	3.8	1.2	1.1	0.7	0.7	1.3	0.7	0.5	0.6	0.4	0.7	0.4
	K4	1.36	1.25	1.42	1.36	1.15	1.31	0.93	0.89	0.93	0.86	1.01	2.5	2.3	2	1.7	1.8	0.9	1.3	1.4	1.3	1.6	0.83
	K5	2.68	2.56	2.79	2.36	2.26	2.36	1.68	1.99	1.99	1.68	1.99	2.9	2.8	2.4	2.3	2.6	1.4	1.6	1.7	1.4	1.8	0.83
	K6	3.75	3.75	3.75	3.45	3.04	3.3	2.91	2.91	2.68	2.56	2.56	3.5	3.6	3	3.6	3.7	2.2	2.7	2.7	1.9	2.8	1.49
	K7	4.08	4.08	4.26	3.91	3.91	3.75	3.17	3.3	3.45	3.3	3.3	4.1	4.6	4	3.9	5.4	3	3.1	3.3	2.6	2.9	1.76
	K9	4.26	4.63	4.83	4.26	5.48	5.26	4.63	4.63	4.26	4.44	4.26	5	4.8	4.6	5.4	6.1	3.7	4.6	4.4	3.7	5.4	3.14

Note: K1–K9 are the Numbers of the sampling outlet from top to bottom of the soil column.

Table S4. F^- concentrations of the water samples collected from the soil column leaching experiment.

Time (h)		0	8	24	36	60	84	106	130	154	178	202	226	250	274	298	322	346	370	394	442	490	538	586
F^- concentration (mg/L)	K1-1	1.4	0.4	0.4	0.4	0.4	0.5	0.4	0.8	0.39	0.38	0.38	0.43	0.38	0.39	/	/	0.38	/	0.37	/	/	/	/
	K1-2	1	0.7	0.7	0.5	0.7	1	0.6	1.1	0.52	0.76	0.59	0.59	0.52	0.52	0.76	0.46	0.48	0.57	0.46	0.54	0.36	0.34	0.4
	K1-3	1.6	1	1.2	1.4	1.7	2.2	1.7	1.9	1.46	1.4	1.46	1.58	1.4	1.46	1.52	1.64	1.4	1.4	1.34	1.23	1.28	1.25	1.3
	K1-4	2.9	1.9	2.3	2.7	3.2	3.9	2.6	3.4	2.8	2.68	2.91	3.03	2.58	2.8	2.8	3.16	2.68	2.68	2.69	2.58	2.69	2.74	2.9
	K2-1	1.2	1	1.5	/	/	/	/	/	/	/	/	/	/	/	/	/	/	/	/	/	/	/	/
	K2-2	1.6	1.1	1.1	1.8	2	2.6	1.8	2.3	2.02	1.86	2.02	2.1	1.71	1.94	1.94	1.94	1.78	1.71	1.78	1.51	1.71	1.54	1.7
	K2-3	1.5	0.9	2.4	1.4	1.6	2	1.5	1.9	1.64	1.58	1.64	1.78	1.58	1.64	1.64	1.86	1.58	1.58	1.64	1.71	1.78	1.82	1.8
	K2-4	3.1	2	1.7	3	3.6	4.4	3.4	4.2	3.43	3.57	3.72	4.21	3.57	3.88	3.88	4.21	3.72	3.72	3.74	3.9	4.23	4.31	4.5
	K3-1	1.2	/	/	/	/	/	/	/	/	/	/	/	/	/	/	/	/	/	/	/	/	/	/
	K3-2	2	1.3	1.4	1.8	1.9	2.5	1.9	2.3	1.86	1.86	2.02	2.1	1.94	2.02	2.02	2.19	1.94	2.02	2.02	2.02	2.1	2.23	2.3
	K3-3	1.8	1.1	2.4	1.7	1.9	2.2	1.7	2.1	1.64	1.71	1.86	1.94	1.71	1.86	1.86	2.02	1.86	1.86	1.94	1.94	2.02	2.14	2.2
	K3-4	3.1	1.9	1.5	3	3.4	4.2	3.1	4	3.03	3.03	3.57	3.72	3.16	3.57	3.57	3.88	3.43	3.72	3.74	3.74	4.06	4.31	4.5
	K4-1	/	/	/	/	/	/	/	/	/	/	/	/	/	/	/	/	/	/	/	/	/	/	/
	K4-2	/	/	/	/	/	/	/	/	/	/	/	/	/	/	/	2.38	1.94	2.02	2.02	2.1	2.28	2.32	2.3
	K4-3	/	/	/	/	/	/	/	/	/	/	/	/	/	/	/	2.38	2.02	1.94	1.94	2.02	2.02	2.05	2.1
	K4-4	/	/	/	/	/	/	/	/	/	/	/	/	/	/	/	6.87	6.07	5.6	5.89	5.65	5.89	5.99	5.8
	K5-1	2.1	1.2	/	/	/	/	/	/	/	/	/	/	/	/	/	/	/	/	/	/	/	/	/
	K5-2	2	1.2	1.5	1.9	2	2.6	2	2.4	1.94	1.94	2.1	2.1	1.94	2.02	2.1	2.02	1.94	2.02	2.1	2.1	2.28	2.32	2.3
	K5-3	1.9	1.3	1.6	1.8	1.9	2.4	1.8	2.2	1.94	1.94	2.1	2.19	1.94	2.1	2.19	2.28	2.02	2.02	2.02	2.1	2.28	2.32	2.4
	K5-4	2.6	1.9	2.3	1.9	2	2.7	2.1	2.3	1.94	1.94	2.02	2.19	2.02	2.1	2.1	2.1	1.94	2.02	2.1	2.1	2.48	2.32	2.4
	K0	/	/	/	2.6	2.6	2.8	2.2	2.8	2.38	2.68	2.38	2.58	2.47	2.68	2.58	2.47	2.58	2.58	2.8	2.58	2.48	2.74	2.9

Note: K0–K5 are the Numbers of the sampling outlet from top to bottom of the soil column.

Table S4. Cont.

Time (h)		634	682	730	778	826	874	922	970	1018	1066	1114	1162	1282	1402	1522	1782	2022	2262	2502	2742	2982	3222
F^- concentration (mg/L)	K1-1	/	/	/	/	/	/	/	/	/	/	/	0.33	0.24	0.24	0.36	0.39	0.29	0.25	0.25	0.18	0.22	0.26
	K1-2	0.4	0.3	0.5	0.5	0.4	0.4	0.3	0.3	0.32	0.34	0.38	0.34	0.3	0.33	0.4	0.39	0.36	0.34	0.3	0.25	0.33	0.38
	K1-3	1.1	1	1	1.1	1.1	1.1	1	1	1	1.05	1.05	1	0.88	0.84	1.03	0.89	0.82	0.82	0.71	0.56	0.61	0.65
	K1-4	2.2	2.2	2.2	2.2	2.2	2.1	2.2	2	2.07	2.16	2.16	2.07	1.9	1.82	2.16	1.92	1.84	2	1.58	1.29	1.3	1.38
	K2-1	/	/	/	/	/	/	/	/	/	/	/	/	/	/	/	/	/	/	/	/	/	/
	K2-2	1.3	1.3	1.3	1.3	1.3	1.2	1.2	1.2	1.24	1.29	1.24	1.19	1.24	1.09	1.43	0.89	1.01	1.15	0.81	0.61	0.82	0.71
	K2-3	1.5	1.5	1.5	1.4	1.5	1.4	1.3	1.3	1.29	1.41	1.35	1.29	1.14	1.05	1.26	1.06	1.06	1.11	0.89	0.69	0.7	0.71
	K2-4	3.6	3.8	3.8	3.6	3.6	3.5	3.5	3.5	3.61	3.45	3.61	3.61	3.31	3.04	3.7	3.08	3.21	3.31	2.56	2.03	2.05	2
	K3-1	/	/	/	/	/	/	/	/	/	/	/	/	/	/	/	/	/	/	/	/	/	/
	K3-2	1.8	1.8	1.8	1.8	1.8	1.7	1.8	1.8	1.82	1.82	1.75	1.82	1.54	1.54	1.49	1.31	1.42	1.55	1.14	0.89	0.82	0.8
	K3-3	1.8	1.8	1.8	1.7	1.7	1.7	1.6	1.7	1.75	1.82	1.82	1.82	1.75	1.67	1.99	1.77	1.76	1.92	1.55	1.24	1.19	1.17
	K3-4	3.3	3.3	3.5	3.5	3.8	3.5	3.5	3.2	3.45	3.45	3.45	3.45	4.1	2.91	3.27	2.83	2.7	3.05	2.35	1.87	1.81	1.84
	K4-1	/	/	/	/	/	/	/	/	/	/	/	/	/	/	/	/	/	/	/	/	/	/
	K4-2	1.9	1.9	1.9	1.9	2	1.8	1.8	1.8	1.75	1.82	1.82	1.82	1.75	1.67	1.91	1.49	1.49	1.76	1.35	1.05	0.89	0.91
	K4-3	1.7	1.8	1.8	1.8	1.8	1.8	1.7	1.7	1.67	1.75	1.67	1.67	1.6	1.54	1.83	1.62	1.76	1.84	1.47	1.14	1.14	1.17
	K4-4	4.8	4.6	5	4.8	5	4.8	4.5	4.5	4.46	4.46	4.46	4.46	4.1	3.76	4.19	3.35	3.07	3.18	2.52	1.95	1.81	1.77
	K5-1	/	/	/	/	/	/	/	/	/	/	/	/	/	/	/	/	/	/	/	/	/	/
	K5-2	1.8	1.8	1.8	1.7	1.8	1.7	1.7	1.8	1.75	1.82	1.75	1.82	1.75	1.67	1.99	1.62	1.55	1.76	1.39	1.05	1.01	0.91
	K5-3	2	1.8	1.8	1.8	1.8	1.7	1.8	1.7	1.9	2.07	2.16	1.9	1.82	1.67	2.07	1.69	1.69	1.84	1.39	1.05	1.01	0.87
	K5-4	2	1.9	1.8	1.8	1.8	1.8	1.7	1.9	1.98	2.07	2.07	2.07	1.9	1.82	2.35	2.01	2	2	1.66	1.29	1.24	1.17
	K0	2.2	2.2	2.1	2.1	2.1	2	1.9	2	1.98	2.07	2.46	2.07	2.16	1.98	2.25	2.1	1.92	2.08	1.59	1.24	1.14	1.08

Note: K0–K5 are the Numbers of the sampling outlet from top to bottom of the soil column.
